# Insertion of a ligand to HER2 in gB retargets HSV tropism and obviates the need for activation of the other entry glycoproteins

**DOI:** 10.1371/journal.ppat.1006352

**Published:** 2017-04-19

**Authors:** Biljana Petrovic, Tatiana Gianni, Valentina Gatta, Gabriella Campadelli-Fiume

**Affiliations:** Department of Experimental, Diagnostic and Specialty Medicine, University of Bologna, via S. Giacomo 12, Bologna, Italy; Icahn School of Medicine at Mount Sinai, UNITED STATES

## Abstract

Herpes simplex virus (HSV) entry into the cells requires glycoproteins gD, gH/gL and gB, activated in a cascade fashion by conformational modifications induced by cognate receptors and intermolecular signaling. The receptors are nectin1 and HVEM (Herpes virus entry mediator) for gD, and αvβ6 or αvβ8 integrin for gH. In earlier work, insertion of a single chain antibody (scFv) to the cancer receptor HER2 (human epidermal growth factor receptor 2) in gD, or in gH, resulted in HSVs specifically retargeted to the HER2-positive cancer cells, hence in highly specific non-attenuated oncolytic agents. Here, the scFv to HER2 was inserted in gB (gB_HER2_). The insertion re-targeted the virus tropism to the HER2-positive cancer cells. This was unexpected since gB is known to be a fusogenic glycoprotein, not a tropism determinant. The gB-retargeted recombinant offered the possibility to investigate how HER2 mediated entry. In contrast to wt-gB, the activation of the chimeric gB_HER2_ did not require the activation of the gD and of gH/gL by their respective receptors. Furthermore, a soluble form of HER2 could replace the membrane-bound HER2 in mediating virus entry, hinting that HER2 acted by inducing conformational changes to the chimeric gB. This study shows that (i) gB can be modified and become the major determinant of HSV tropism; (ii) the chimeric gB_HER2_ bypasses the requirement for receptor-mediated activation of other essential entry glycoproteins.

## Author summary

Herpes simplex virus encodes an entry apparatus made of the glycoproteins gD, gH/gL and gB. gD is the major determinant of HSV tropism. Receptor-induced modifications to gD and gH/gL activate in a cascade fashion gB, the conserved fusogenic glycoprotein across the *Herpesviridae* family. In herpesviruses other than HSV, but not in HSV, gB also contributes to determine the virus tropism. We took advantage of retargeting studies to investigate the process of HSV glycoprotein activation, and the specific roles played by the glycoproteins. When a heterologous ligand is engineered in gB, the virus tropism is retargeted to the ligand receptor. gB becomes the major determinant of HSV tropism, and does not any longer need the receptor-mediated activation of glycoproteins gD and gH/gL.

## Introduction

Herpes simplex virus encodes a multipartite entry apparatus made of four essential glycoproteins, named gD, the heterodimer gH/gL and gB, with distinct functions [1–4]. gD, whose structure includes an Ig-folded core with extensions, serves as a typical receptor-binding glycoprotein, and the major determinant of HSV tropism [5–7]. The heterodimer gH/gL is a multidomain protein, with no structural resemblance to any known protein [8–10]. gB is a trimer with structural features typical of viral fusion glycoproteins [11–13]. gH/gL and gB form the conserved fusion apparatus across the *Herpesviridae* family. The quartet assembles in complexes [14, 15, 16–18]. Contact regions among the glycoproteins were identified [10,17–20]. The system of receptors for the quartet of glycoproteins appears to be more and more complex, and affects the process of glycoprotein activation at virus entry. gD interacts with three alternative receptors, nectin1, HVEM, and modified heparan sulphate [21–24]. gH/gL interact with the αvβ subfamily of integrins [25,26]. αvβ6 and αvβ8 are required for entry, in that their depletion, or block with antibodies, results in block to virus infection [26]. Three co-receptors for gB were reported. They are PILRα (paired immunoglobulin-like type 2 receptor-alpha), myelin associated glycoprotein, and isoforms IIA and IIB of non-muscle myosin heavy chain [27–30]. Little is known about the role they play in HSV entry. In particular, there is no evidence that they contribute to define the host range of the virus. PILRα was reported to be expressed, and possibly to play a role in HSV infection of monocytes, a cell type not usually targeted by HSV [27]. The effect, if any, of depleting this receptor in epithelial cells, the targets of wt-HSV *in vivo*, was not investigated. The lack of contribution of gB receptors to overall viral tropism is highlighted by the finding that abrogation of the gD interaction with one of its receptors abrogates virus entry in virtually any cell.

The current model of HSV entry envisions that the four glycoproteins switch from an inactive to a fusion-active conformation [2–4,31]. Although numerous steps in this model remain to be elucidated, it is well documented that activation is triggered by the gD binding to one of its alternative receptors, and then propagates to gH/gL, and finally to gB in a cascade fashion. The conformational changes to gH/gL are induced upon transmission of a signal from receptor-bound gD, and upon interaction with one of the two integrins, and was documented as displacement of gL from the heterodimer [26,32]. The displacement only occurs if all the viral and cellular components of the entry apparatus are present, and is prevented by a MAb to gH with strong neutralizing activity, supporting the view that it is part of the process of gH activation [32].

The HSV glycoproteins are of interest in the design of oncolytic HSVs. Recently, this field received much attention upon Federal Drug Administration and European Medicines Agency approval of the oncolytic HSV, originally named Oncovex^GM-CSF^, for the treatment of metastatic melanoma [33,34]. For this virus, as well as numerous oncolytic viruses, cancer specificity has been achieved at the expense of virulence [35,36]. In essence, they carry deletions or mutations in genes involved in contrasting the innate response to the virus, and take advantage of the fact that cancer cells mount a very weak response to them [37–39]. The drawback is that these viruses are attenuated and may replicate poorly [37]. The strategy pursued by other laboratories, including ours, is to develop non-attenuated oncolytic HSVs, by retargeting the HSV tropism to cancer-specific receptors [40–42]. The initial studies identified gD as the glycoprotein to be modified in order to readdress virus tropism to a receptor of choice, and detarget from the natural gD receptors [41]. In our studies, the selected receptor was HER2 (human epidermal growth factor receptor 2), a member of the EGFR (epidermal growth factor receptor) family, overexpressed in about 25–30% of breast and ovary cancers, as well as in stomach, lung and other cancers [43]. Retargeting was achieved by engineering in gD a single chain antibody (scFv) to HER2, derived from trastuzumab, and by appropriate deletions in gD, which remove critical residues for interaction with HVEM and nectin1 [44–47]. Recent studies from our laboratory showed that also gH can be a tool for retargeting [48]. Thus, the insertion of a scFv to HER2, combined with an appropriate deletion in gD, lead to a HSV fully retargeted to HER2 through gH.

Here, we asked whether gB is a suitable glycoprotein for retargeting. We engineered the scFv to HER2 between AA 43 and 44 of gB, thus generating R-903. By further deletion of AA 6–38 in gD, the recombinant could be detargeted from natural receptors. The retargeting to HER2 *via* gB was unexpected, since gB is the fusogenic glycoprotein, and was not known to be a determinant of HSV tropism. Inasmuch as the scFv to HER2 mediates entry when engineered in gD, gH, or gB we asked how can a same ligand, engineered in one or the other of the three glycoproteins—gD, gH or gB—enable entry through the HER2 receptor.

## Results

### Engineering of recombinants carrying scFv to HER2 in gB

**Fig 1:**
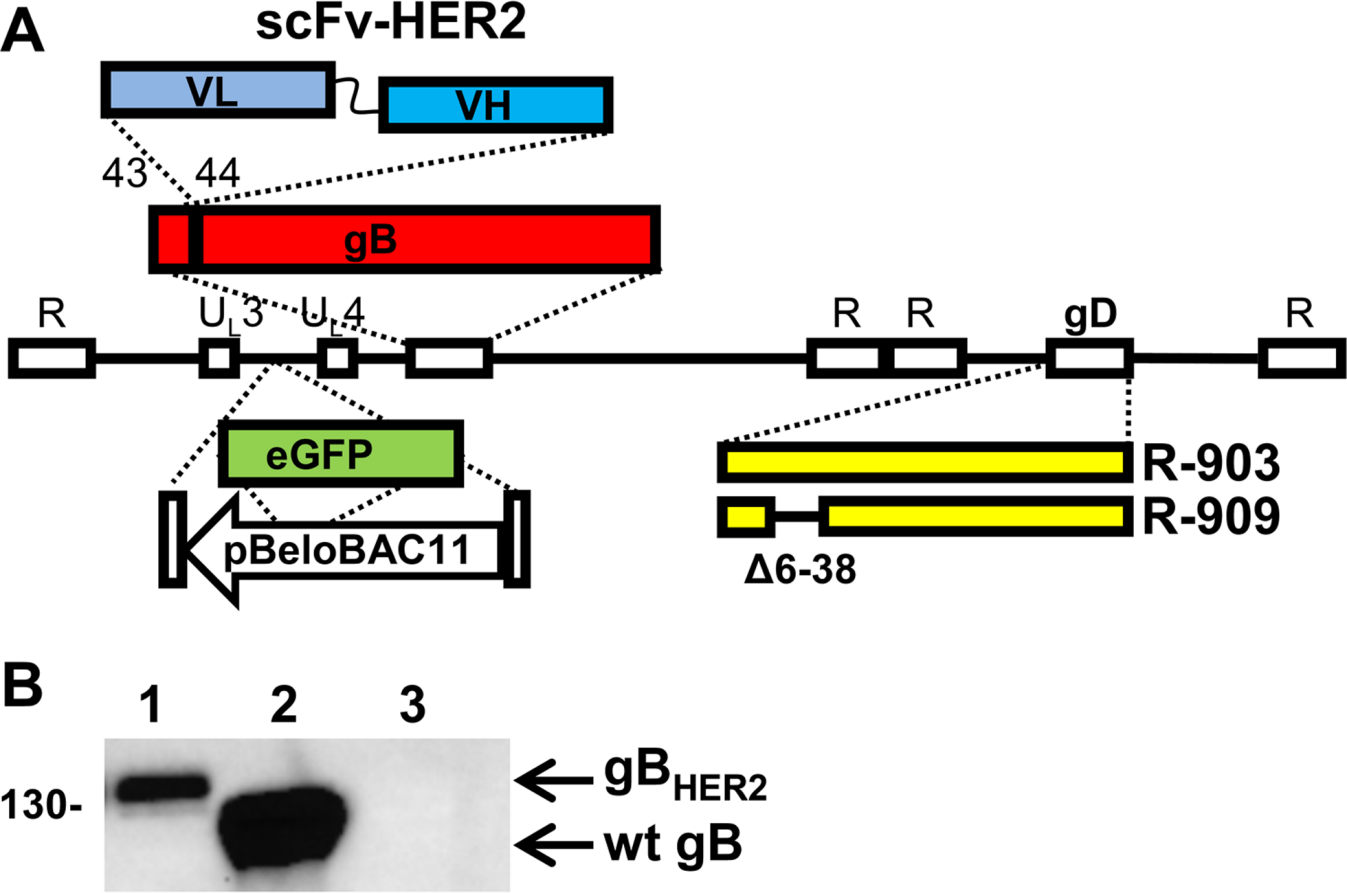
(A) Genome arrangement of recombinants R-903 and R-909. The HSV-1 genome is represented as a line bracketed by repeats (R). The Lox-P-bracketed BAC sequence and eGFP fluorescent marker are inserted in the intergenic region U_L_3-U_L_4. R-903 carries the insertion of scFv-HER2, with a downstream 12 Ser-Gly linker, between AA 43–44 of gB. R-909 is the same as R-903; in addition, it carries the deletion of AA 6–38 from mature gD for detargeting purpose. (B) R-909 expresses the chimeric gB_HER2_ glycoprotein. Lysates of SK-OV-3 cells infected with R-909 (lane 1), or the wt R-LM5 (lane 2) (3 PFU/cell) were subjected to PAGE, and immunoreacted with MAb H1817 to gB [53]. Lane 3 indicates uninfected SK-OV-3 cells. Arrows point to the migration position of chimeric gB_HER2_ and of wt-gB. Figure to the left indicates the migration position of M_r_ markers, expressed in kDaltons.

**Fig 2:**
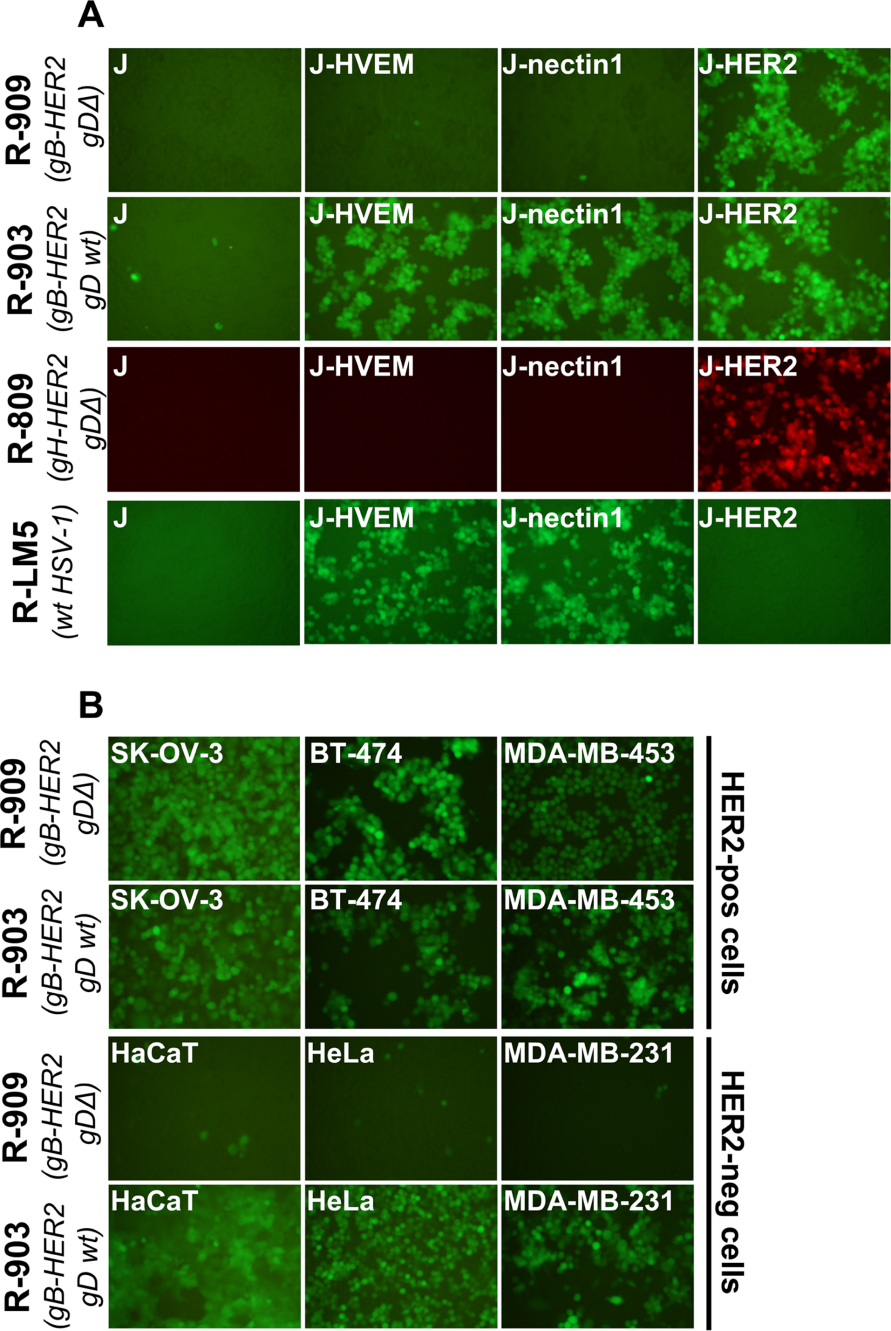
R-909 specifically infects HER2-pos cells. (A) Both the R-909 and the non-detargeted R-903 infect cells that express HER2 as the sole receptor (J-HER2 cells). J cells express no receptor for wt-HSV. J-HER2, J-nectin1 and J-HVEM express only the indicated receptor. The pattern of infection of R-909 is similar to that of R-809, and differs from that of R-LM5. (B) R-909, but not R-903, specifically infects HER2-pos, and fails to infect the HER2-neg cancer cells. In all panels, the indicated cells were infected at 3 PFU/cell. Infection was monitored at 24 h by fluorescence microscopy.

The scFv to HER2 was engineered in gB between AA 43–44, thus generating R-903 (Fig 1A). This position is known to accept the heterologous ligand green fluorescent protein (GFP) [49]. The R-909 recombinant was derived from R-903 by deletion of AA 6–38 in gD, for detargeting from the natural gD receptors, HVEM and nectin1 (Fig 1A) [45]. Both recombinants carry the Lox-P bracketed BAC sequence and the eGFP (enhanced green fluorescent protein), cloned in the intergenic UL3-UL4 region. The presence of the scFv insert was verified by sequencing the ORF, and by sodium dodecyl sulphate-polyacrylamide gel elecctrophoresis (SDS-PAGE) and immunoblotting. As expected, gB from R-909 exhibited a lower electrophoretic mobility than wt-gB present in the R-LM5 recombinant (Fig 1 B). The latter recombinant carries the BAC and eGFP sequences and is otherwise wt (see Fig 2 A for its tropism) [45].

### R-903 and R-909 infect cells that express HER2 as the sole receptor, use HER2 as portal of entry, and do not necessitate of a gD receptor

The modifications to tropism were assayed in J cells which transgenically express the receptor of choice, HVEM, nectin1, or HER2. J cells are negative for HSV gD receptors and cannot be infected by wt-HSV [21]. The cells were infected with R-903 and R-909 recombinants, and scored by fluorescence microscopy. Fig 2A shows that R-903 and R-909 infected J-HER2 cells, implying that they were retargeted to HER2. R-903 also infected J-HVEM and J-nectin1 cells, as expected, given that it encodes a wt-gD. In contrast, R-909 failed to infect through the gD receptors, in agreement with the AA 6–38 deletion in gD. The tropism of R-909 was essentially similar to that of R-809 (previously named R-VG809), retargeted to HER2 by insertion of scFv in gH, and detargeted from gD receptors [48]. As expected, the wt R-LM5 infected J-nectin1 and J-HVEM cells and failed to infect J-HER2 cells. For a summary of nomenclature and properties of viruses employed in this study, see Table 1. R-909 was further assayed for ability to infect HER2-pos (SK-OV-3, BT-474, MDA-MB-453) and HER2-neg HeLa, MDA-MB-231 cancer cells, and the keratinocytic cell line HaCaT. The non-detargeted R-903 was included as control. Fig 2B shows that R-909 infected the HER2-pos cells, but failed to infect the HER2-neg cells. By contrast, R-903 infected both sets of cells. The results strengthen the conclusion that R-909 is retargeted to HER2 *via* gB and detargeted from natural gD receptors.

**Table 1:** Summary of genotypic modifications and tropism of recombinants.

Virus	Viral gene in which scFv-HER2 is inserted	Additional genotypic modification	Retargeting to HER2	Detargeting from nectin1/HVEM	Ref.
R-903	gB	none	+	-	This paper
R-909	gB	gD_Δ6–38_	+	+	This paper
R-809*	gH	gD_Δ6–38_	+	+	[48]
R-LM113	gD	gD_Δ6–38_	+	+	[45]
R-LM249	gD	gD_Δ61–218_	+	+	[46]
R-LM5	none	none	-	-	[45]

*, previously named R-VG809

Three main conclusions can be drawn from this set of experiments. First, the insertion of a scFv in gB modifies HSV tropism. This was a surprising result since gB is known for its ability to carry out virion-cell fusion, but not as determinant of HSV tropism. Second, the finding that R-909 infected J-HER2 cells, but not J cells, suggests that the scFv in gB enabled gB activation upon interaction with HER2, and rules out that gB activation occurred independently of the interaction with HER2. Lastly, the infection of J-HER2 cells with R-909 occurs in the absence of a gD receptor, or with a deleted gD unable to bind its natural receptor. Essentially, it is independent of receptor-mediated gD activation. These properties imply that the cascade of glycoprotein activation that ultimately leads to gB activation does not occur in a canonical manner in R-909, i.e. starting from receptor-bound gD and *via* transmission to gH/gL and then to gB.

### Replication and killing activity of R-909

**Fig 3:**
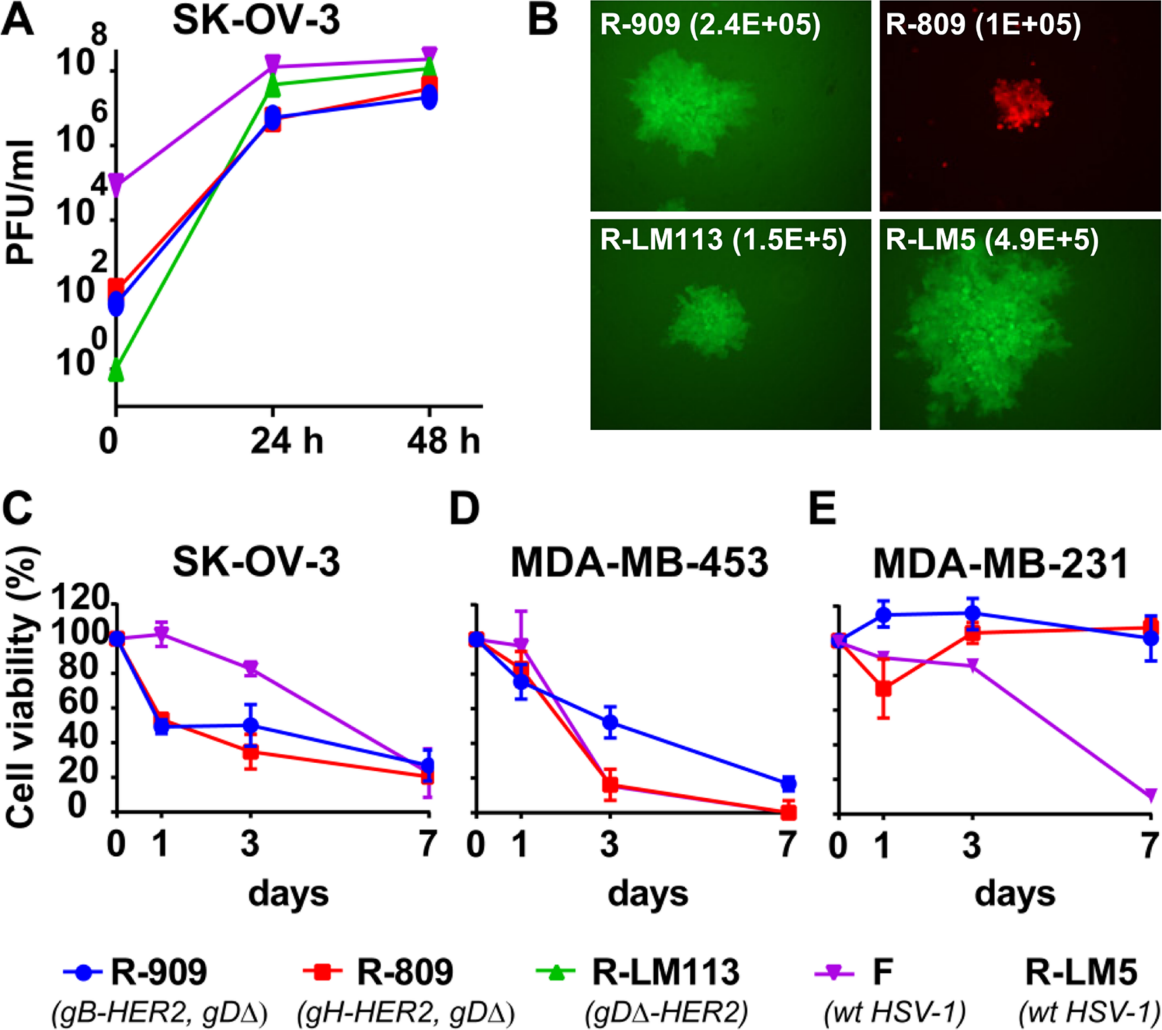
Extent of R-909 replication in SK-OV-3 cells, plaque size and ability to kill cells. (A) SK-OV-3 cells were infected with R-909, with R-809, R-LM113, wt HSV-1(F), as controls, at 0.1 PFU/cell, and harvested at the indicated times (h) after infection. Progeny virus was titrated in SK-OV-3 cells. (B) Relative plaque size of R-909, and of R-809, R-LM113, wt R-LM5, as controls, in SK-OV-3 cells. Figures in brackets represent the average size of 10 plaques, in pixels. (C-E) Killing ability of R-909, R-809 and HSV-1(F) for the HER2-pos SK-OV-3 and MDA-MB-453 cells, and absence of killing ability for the HER2-neg MDA-MB-231 cancer cells. SK-OV-3 and MDA-MB-453 cells were infected at 2 PFU/cell and MDA-MB-231 cells were infected with R-909 and R-809 at 0.1 PFU/cell. MDA-MB-231 cells were infected with wt HSV-1(F) at 0.05 PFU/cell. Cell viability was quantified by alamarBlue assay at the indicated days after infection. Results are the average of at least two independent experiments, each of which carrying quadruplicate samples ± S.D.

Fundamental properties for any candidate oncolytic virus are the extent of replication and the ability to kill the infected cells. SK-OV-3 cells were infected with R-909 at 0.1 PFU/cell. The gH-retargeted R-809, the gD-retargeted R-LM113, and the wt HSV-1(F) were included for comparison. Virus yields were determined at 0 time (3 h), 24 and 48 h after infection. Fig 3A shows that R-909 grew to titers very similar to those of R-809, and less than one log lower than those of the gD-retargeted R-LM113. All retargeted viruses replicated between 1–2 log less than the parental HSV-1(F). Fig 3B shows that plaques from R-909 were bigger in size relative to R-809 and R-LM113; all were smaller relative to R-LM5.

The killing ability of R-909 for the HER2-pos MDA-MB-453 and SK-OV-3 cancer cells is reported in Fig 3C–3E. Cytotoxicity of R-909 was very similar to that of R-809 and HSV-1(F), especially at 7 days after infection, and ranged from 70 to 90% at 7 days after infection for the HER2-positive SK-OV-3 and MDA-MB-453 cells. R-909 and R-809 failed to kill the HER2-neg MDA-MB-231 cancer cells (Fig 3E), consistent with failure to infect them, whereas the wt HSV-1(F) killed about 90% at 7 days after infection (Fig 3E). Cumulatively, the results show that retargeting through gB confers very similar properties as retargeting through gH, in terms of virus growth, plaque size and killing ability.

### R-909 uses HER2 as portal of entry, and infection is inhibited by neutralizing MAbs to gD, gH, gB

**Fig 4:**
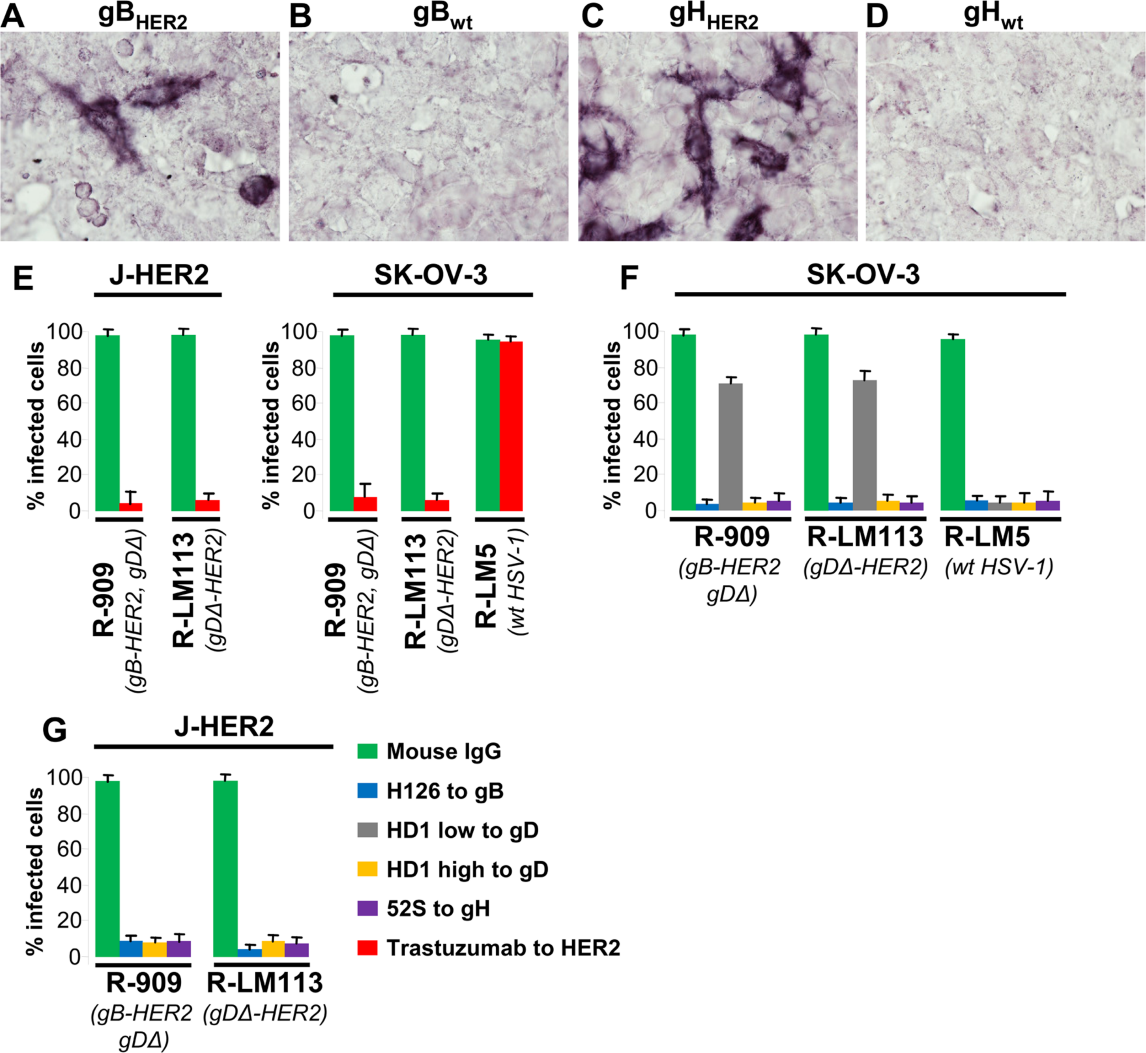
gB_HER2_ and gH_HER2_ interact with a truncated soluble form of HER2, and infection of R-909 is dependent on HER2 and is inhibited by antibodies to gD, gH/gL, gB. (A-D) DNA encoding wt gH/gL (D), gH_HER2_/gL (C), wt gB (B) or gB_HER2_ (A), was transfected into 293T cells. 24 h later, cells were fixed with paraformaldehyde, and then reacted with a truncated soluble form of HER2 (E) J-HER2 and SK-OV-3 cells were infected with R-909, R-LM113, R-LM5. Cells were pretreated with trastuzumab (28 μg/ml) or control mouse IgGs (28 μg/ml). Extent of infection was quantified 24 h later by flow cytometry, and expressed as percentage relative to cells infected with or untreated cells. Each value represents the average of three independent experiments ± S.D. (F-G) R-909, R-LM113, and the wt-R-LM5 were preincubated with MAbs HD1 to gD (low, 1.5 μg/ml; high, 30 μg/ml), MAb 52S to gH/gL (ascites fluid 1:25), or MAb H126 to gB (ascites fluid 1:2000) for 1 h at 37°C, and then allowed to infect SK-OV-3 (F) or J-HER2 (G) cells. Extent of infection was quantified 24 h later by flow cytometry, and expressed as percentage relative to cells infected with untreated virus. Each value represents the average of three independent experiments ± S.D.

To verify that R-909 infection occurs through the interaction of the chimeric gB (gB_HER2_) with HER2, we verified whether gB_HER2_ binds HER2, and whether R-909 infection was inhibited by trastuzumab, the MAb to HER2 from which the scFv was derived [50]. For the binding assay, we cloned gB_HER2_ from R-909, gH_HER2_ from R-803. 293T cells were transfected with gB_HER2_, gH_HER2_, or their wt counterpart, and reacted with a soluble truncated form of HER2. Fig 4A and 4C show that the chimeric gB_HER2_, and gH_HER2_ reacted with soluble HER2, whereas the wt-gB and wt-gH did not. This result shows that the binding of gB_HER2_, or gH_HER2_ to HER2 occurs in the absence of the other glycoproteins. For the infection assay, we infected J-HER2 or SK-OV-3 cells in the presence of trastuzumab. A wt HSV was not included as it does not infect J-HER2 cells (see Fig 2A and references [44] and [45]). Trastuzumab blocked entry of R-909 and R-LM113, but not of R-LM5 (Fig 4E), indicating that R-909 uses HER2 as a portal of entry.

This finding and the above implication that R-909 did not require the canonical cascade of glycoprotein activation triggered by the gD interaction with nectin1 or HVEM prompted us to further characterize the entry process of R-909. We asked whether R-909 infection was inhibited by neutralizing antibodies to gD, gH and gB. R-LM113, and R-LM5 (see, Table 1) were included for comparison. Virions were preincubated with MAbs to gD (HD1), to gH (52S), or to gB (H126) [51–53]. All antibodies inhibited R-909 infection (Fig 4F and 4G); at low concentrations MAb HD1 failed to inhibit entry of R-909 and R-LM113, as noted earlier [48]. The results indicate that infection with R-909 requires the essential glycoproteins gD, gH and gB.

Altogether, current and previous findings that HER2 can mediate HSV entry once the scFv to HER2 is inserted in gD, in gH, or in gB, raise the question as to the mechanism by which HER2 mediates entry of the three sets of recombinants. Subsequent experiments were finalized to address this question, and were conducted by comparing R-909 to the gH-retargeted R-809 and to the gD-retargeted R-LM113 and R-LM249.

### Role of gD in the cell-cell fusion mediated by retargeted gB and by retargeted gH

**Fig 5:**
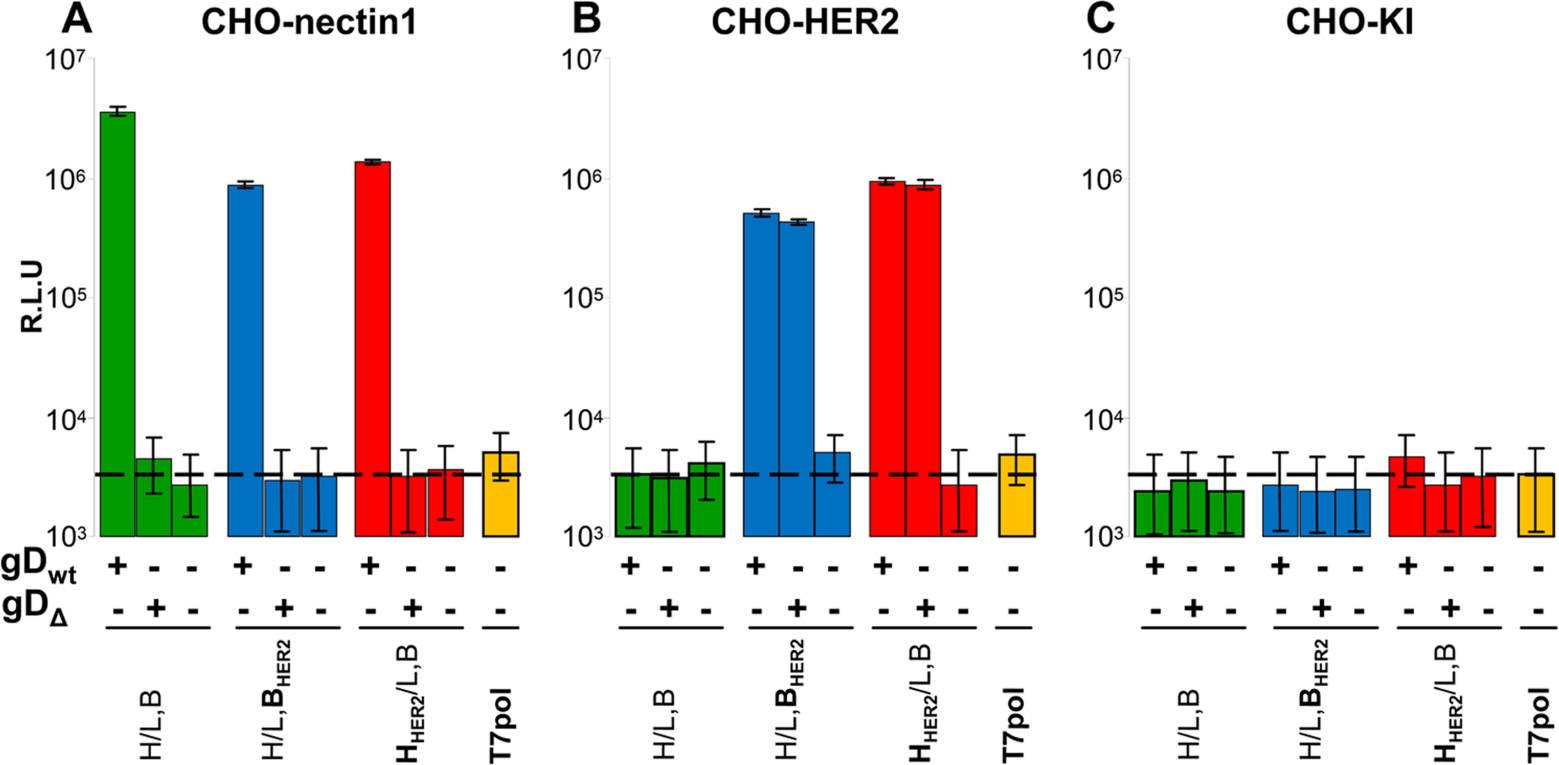
gD is required to promote cell-cell fusion by HER2-retargeted gB_HER2_ or gH_HER2_. (A-C) Donor CHO cells were transfected with the indicated mixtures of the following glycoproteins: wt-gD (gD_wt_), gD_Δ6–38_ (gD_Δ_), wt-gH (H), HER2-retargeted gH (H_HER2_), wt-gB (B), HER2-retargeted gB (B_HER2_), wt-gL (L) and T7 polymerase. Target cells were transfected with nectin1 (A), HER2 (B), or no receptor (C) plus T7-driven luciferase. Extent of fusion was expressed as relative luciferase units (R.L.U.). Each column represents the average of triplicate samples ± S.D.

The requirement for gD in infection of R-909 is in apparent contrast with the lack of requirement for receptor-mediated gD activation seen in J-HER2 cells, and may reflect multiple functions of this glycoprotein. Evidence in favour of receptor-independent gD activities in virus infection and cell-cell fusion was provided [42,54,55]. In particular, in addition to the triggering role exerted by receptor-bound gD, gD may play a “structural” role [48]. Inasmuch as the entry glycoproteins assemble in complexes also in the absence of gD receptors [14–18], the complete absence of anyone of the glycoproteins, or their binding to antibodies, may affect the stability/structure/stoichiometry/gymnastics of the complexes [48]. A variety of techniques were employed for detection of the complexes. Often, it was unclear whether the complexes under study were or not fusion-competent, and/or whether they reflected functional complexes in the virion envelope. A surrogate functional technique to infection has been the extensively used cell-cell fusion assay. A key problem in interpreting the results of this assay is that it is not known whether mutant glycoproteins, or specific mixtures, which exhibit a low level activity in the cell-cell fusion, would give rise to infectious or non-infectious viruses. Thus, a very low level of cell-cell fusion (about 4% of the fusion obtained with wt-glycoproteins) was detected in the absence of gD with a partially deleted form of gH, which was interpreted as a partially activated form of gH [56]. Virions carrying such deleted form of gH were not generated. In contrast to gH, virions carrying hyperactive forms of gB in the presence of gD, exhibited no infection in the absence of gD [42]. All in all, a non-triggering, structural role of gD is plausible, but has not been clearly documented so far. The HER2-retargeted gH (gH_HER2_) and gB (gB_HER2_) offered the opportunity to dissect these two functions of gD. We set up a cell-cell fusion assay, whereby the donor cells express the glycoproteins and T7 Polymerase, and the target cells express the receptor—HER2 or nectin1—along with T7 promoter driven luciferase. gB_HER2_ was cloned from R-909, and the gH_HER2_ was cloned from R-803. To mimic the situation in R-909 and R-809 recombinants, we also cloned gD_Δ6–38_. These glycoproteins, or their wt versions, were transfected in appropriate combinations. Fig 5A shows that glycoprotein mixtures which included the gB_HER2_ or gH_HER2_ promoted fusion with CHO-nectin1 cells in the presence of wt-gD. These results imply that gB_HER2_ and gH_HER2_ maintain the fusogenic, or pro-fusogenic, activity of their wt counterparts, although at somewhat reduced extent, in agreement with the infectivity of R-909 and R-809. Importantly, in the absence of gD, the fusion activity dropped by at two-three logs and reached the background level (average value 5 x 10E3 relative luciferase units), suggesting that gD can not be omitted. Fig 5B shows that glycoprotein mixtures which included gB_HER2_, or gH_HER2_ exhibited a significant fusion activity with CHO-HER2 cells, again somewhat lower than that of their wt counterparts with CHO-nectin1 cells. wt-gB and wt-gH did not induce fusion with CHO-HER2 cells. The fusion activity with receptor-negative CHO cells was at the background level (Fig 5C). Thus, the cell-cell fusion assay faithfully mirrored the pattern of infection of HER2-positive cells with R-909 or R-809 virions. Of note, wt-gD could be replaced with gD_Δ6–38_, again mirroring the situation with R-909 and R-809. However, no cell-cell fusion activity was detected in the total absence of gD. Hence, although wt-gD, or gD_Δ6–38_, are not activated by HER2, and therefore do not play a triggering activity, gD can not be omitted. The results provide experimental evidence in favour of a role of gD other than triggering, most likely in favour of its structural role.

### Entry pathway

**Fig 6:**
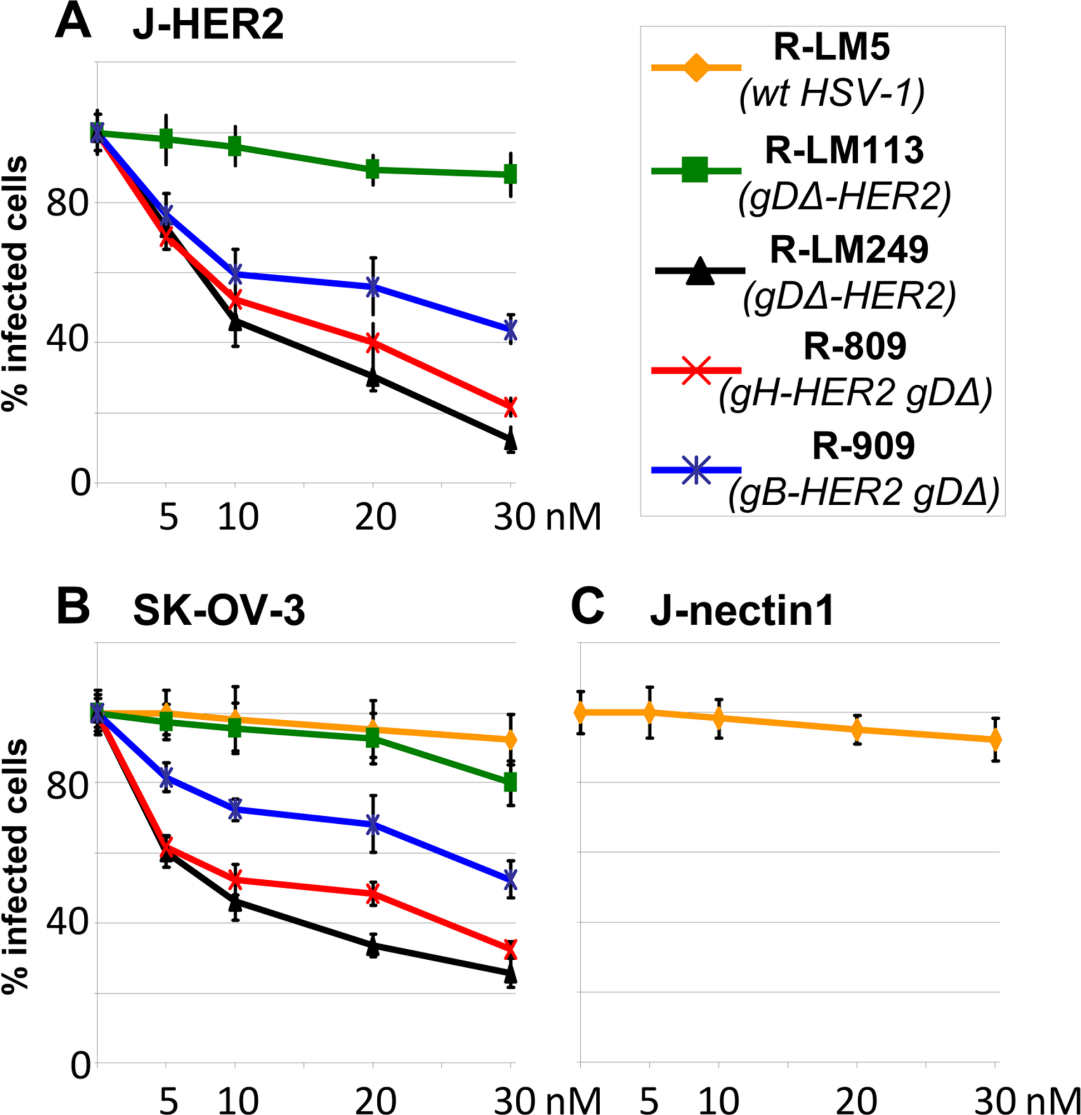
Endocytic entry of R-909 into J-HER2 and SK-OV-3 cells. (A-C) J-HER2 (A), SK-OV-3 (B), or J-nectin1 (C) cells were infected with the indicated viruses, in the presence of increasing nM concentrations of Bafilomycin A (BFLA). The extent of infection of GFP-positive viruses was determined by GloMax Discover System (Promega), and that of R-809 was determined by FACS. Each point represents the average of triplicate samples ± S.D.

Entry of HSVs into the cell occurs in a cell line dependent fashion by fusion at plasma membrane (or with neutral endosomes), or by endocytosis into acid endosomes [57–59]. αvβ3 and αvβ6 integrins intervene as routing factors, and promote the endocytic pathway of entry [26,60]. Thus, infection of J-nectin1 cells with wt-HSV occurs by fusion at plasma membrane, and is not inhibited by bafilomycin A (BFLA) [60]. Infection of J-nectin1 cells overexpressing αvβ3 or αvβ6 integrins is *via* acidic endosomes and inhibited by BFLA. Here, we investigated the pathway of R-909, R-809, R-LM113 and R-LM249 infection of J-HER2 and SK-OV-3 cells. The BFLA inhibition curve shows that infection of J-HER2 cells was decreased with all recombinants, except R-LM113 (Fig 6A). Inhibition of SK-OV-3 cell infection was essentially similar (Fig 6B). In these cells, the entry of wt R-LM5 was not inhibited by BFLA. Even the infection of J-nectin1 cells with R-LM5 was not inhibited by BFLA (Fig 6C), in agreement with earlier work [58]. Altogether, HER2 promoted an acidic endosome pathway of entry for all recombinants, but not for R-LM113. This property is in agreement with promotion of endocytosis by EGFR family members [61]. Why R-LM113 and R-LM249 behave differently is unclear at present. The two viruses differ with respect to the site of scFv insertion, which is N-terminal in R-LM113, and replaces the gD core in R-LM249.

### Entry of R-909 does not require αvβ6 or αvβ8 integrins and does not result in gL displacement from gH/gL heterodimer

**Fig 7:**
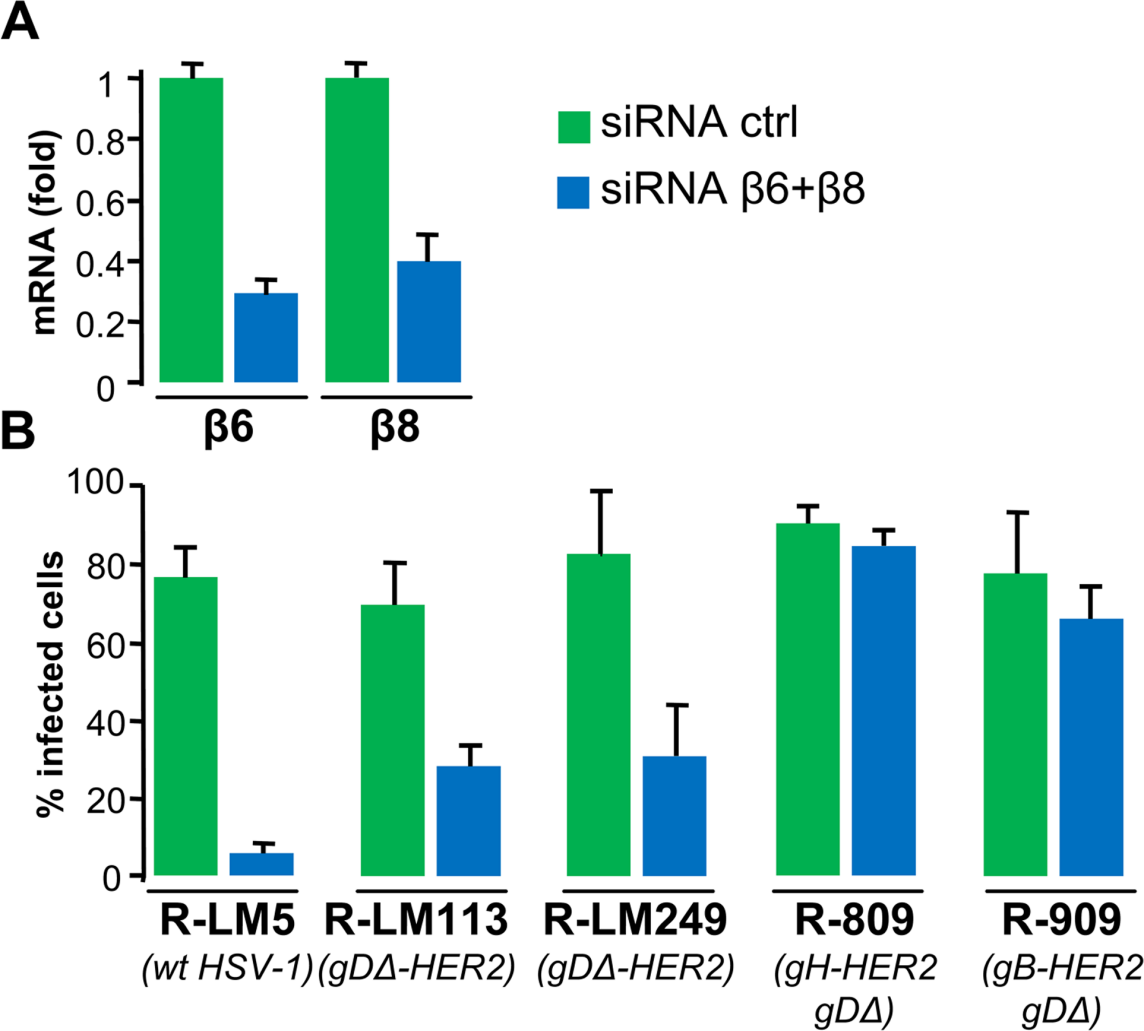
Inhibition of infection in cells depleted of β6 and β8 integrins. (A) Extent of integrins silencing. SK-OV-3 cells were doubly silenced for β6 and β8 integrins (siRNA β6+β8), or mock-silenced (siRNA ctrl). Extent of silencing was measured by qRT-PCR, and expressed as fold decrease relative to siRNA ctrl cells. Each column represents the average of triplicates from two independent experiments ± S.D. (B) Effect of integrins silencing on infection. SK-OV-3 cells silenced with siRNA ctrl, or siRNA β6+β8, were infected with wt R-LM5, R-LM113, R-LM249, R-809 and R-909 (5 PFU/cell), and harvested 24 h later. The extent of infection was quantified by flow cytometry. Each point represents the average of triplicates. Bars show S.D.

Previously, our laboratory showed that αvβ6 or αvβ8 integrins serve as receptors for HSV entry, bind gH and contribute to its activation [26,62]. Hence, αvβ6 or αvβ8 integrins are part of the mechanism of HSV glycoprotein activation that starts with the receptor-bound gD. Here, we tested whether infection with the retargeted viruses R-909, R-809, R-LM113, and R-LM249 requires αvβ6 or αvβ8-integrins. SK-OV-3 cells (which express nectin1, αvβ3, αvβ6 and αvβ8 integrins) were simultaneously depleted of β6 and β8 subunits, or mock depleted (Fig 7A), and then infected. As controls, we used the wt R-LM5. Extent of infection was monitored through eGFP or mCherry. The results summarized in Fig 7B show that infection with R-909 was unaffected by β6 and β8 integrins depletion. By contrast, infection with the wt R-LM5 was almost completely abolished. Infection with the gD-retargeted R-LM113 or R-LM249 was decreased but not abolished in the depleted cells. Interestingly, also the infection with the gH-retargeted R-809 was not decreased by the β-integrins depletion. Thus, the recombinants retargeted through gB, or through gH, exhibited null-to-low requirement for β6 or β8 integrins. In contrast, the wt virus and the gD-retargeted virus exhibited a very high, or high requirement for β6 and β8-integrins. In brief, there appears to be a gradient in terms of integrins-dependence going from wt- and gD-retargeted, to gH-retargeted and to gB-retargeted recombinants.

**Fig 8:**
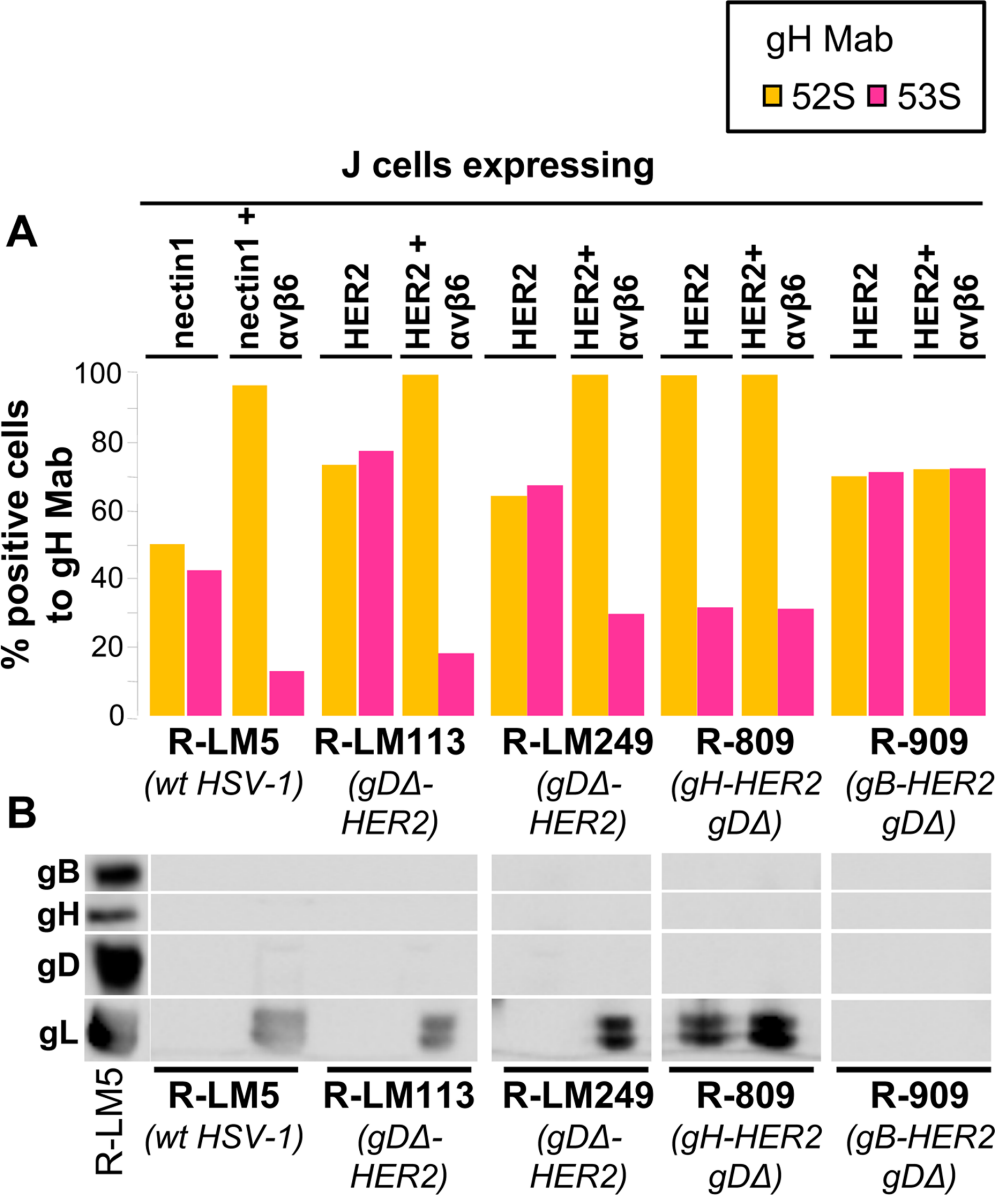
Immunoreactivity of cells carrying absorbed virions to MAbs 52S and 53S to gH, and release of gL in medium. (A) Partially purified virion preparations of wt R-LM5, R-LM113, R-LM249, R-809 and R-909 were absorbed for 30 min at 37°C to J cells expressing nectin1 + αvβ6-integrin (nectin1+αvβ6), HER2 + αvβ6-integrin (HER2+αvβ6), nectin1 alone (nectin1), or HER2 alone (HER2). Cells carrying absorbed virions were then reacted with MAbs 52S (yellow) or 53S (pink) and analyzed by FACS. The percentage of FACS positive cells to each MAb was determined. (B) Release of gL in medium. Immunoreactivity of the media harvested after the 30 min virus absorption to cells (same samples as in A) to PAb gH/gL, MAb gB, and MAb gD. The lanes to the left marked as R-LM5 show the electrophoretic mobility of the indicated HSV glycoproteins.

The ligand to αvβ6 or αvβ8 integrins is gH/gL [26]. Earlier, we showed that integrins induce a conformational change to gH/gL, that results in the displacement of gL from the heterodimer [32]. The displacement occurs at virus attachment/entry, and is prevented under conditions that block virus entry. It only occurs if all the components of the entry apparatus are present, in particular it requires receptor-activated gD as well as gB. Several lines of evidence indicated that it is most likely part of the activation process of gH/gL [32]. The gL displacement can be readily monitored in cell overexpressing αvβ6 integrin, by comparing the reactivity of virions to two anti-gH MAbs. MAb 52S recognizes a gL-independent epitope, and reflects the total quantity of virions absorbed to cells. MAb 53S recognizes a gH epitope that is formed only when gH heterodimerizes with gL (gL-dependent gH epitope) [52]. Once virions attach to cells, the gL displacement is detected as a decrease in MAb 53S reactivity, relative to MAb 52S reactivity [32]. Here, we asked whether the HER2-mediated entry of R-909 entails gL displacement, hence gH activation. J-HER2 or J-HER2 cells expressing αvβ6 integrin (J-HER2+αvβ6) were exposed to R-909 and, for comparison, to R-LM113, R-LM249 and R-809. Fig 8A shows that:

- attachment/entry of R-909 to J-HER2 or J-HER2+αvβ6 cells did not result in a decrease in 53S reactivity relative to 52S reactivity, irrespective of the overexpression of αvβ6 integrin.
- By contrast, attachment/entry of R-LM113 and R-LM249 to J-HER2+αvβ6, but not to J-HER2 cells resulted in a sharp decrease in 53S reactivity, as previously observed for the wt-HSV, herein exemplified by R-LM5.
- Attachment/entry of the gH-retargeted R-809 did result in decrease in 53S reactivity; however, this decrease took place independently of whether αvβ6 was present or absent; hence, it was induced by HER2 and not by integrin.

In the samples where the 53S reactivity was decreased, there was a concomitant release of gL in the medium (Fig 8B). We conclude the following.

- The gL displacement mediated by αvβ6 integrin takes place with R-LM113 and R-LM249; in this case, in contrast to the wt-virus, the gD-activation is mediated by HER2 and not by nectin1.
- The gL displacement takes place with R-809; in this case it is independent of αvβ6-integrin, and is mediated by HER2, which directly targets gH.
- The gL displacement does not take place in R-909, either in the absence or in the presence of αvβ6 integrin. The data argue that attachment/entry of R-909 is independent of β-integrins, does not lead to gL displacement, and therefore the integrin-mediated gH activation does not appear to be a critical step.

### A soluble form of HER2 replaces the membrane-bound HER2 and mediates R-909 infection

**Fig 9:**
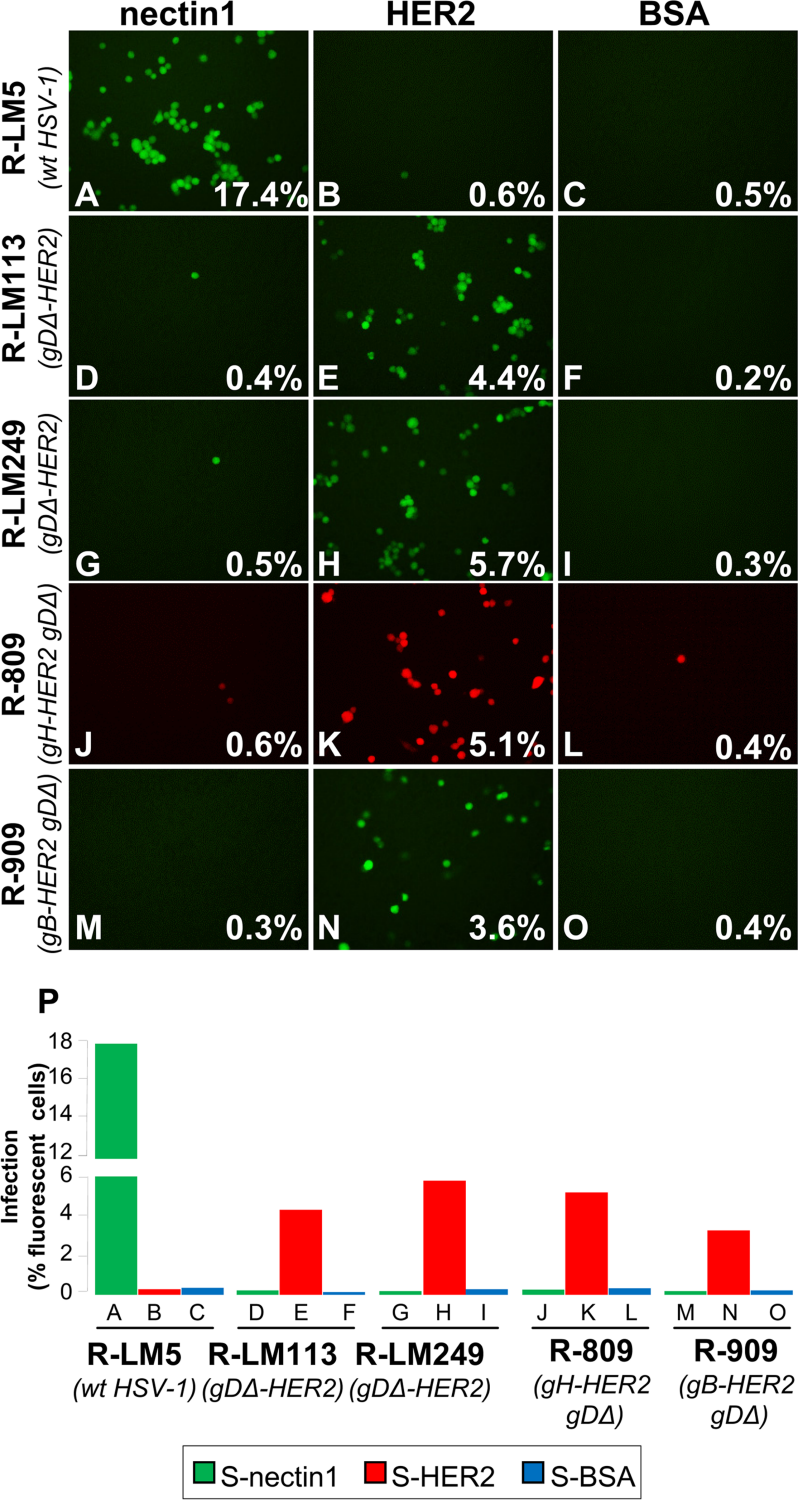
Virus entry mediated by a soluble HER2. (A-O) Receptor-negative J cells were exposed to R-LM5 (A-C), R-LM113 (D-F), R-LM249 (G-I), R-809 (J-L), and R-909 (M-O) for 3 h at 37°C. Soluble form of HER2 (S-HER2) (150 nM) (B, E, H, K, N), nectin1 (S-Nectin1) (150 nM) (A, D, G, J, M), or bovine serum albumin (BSA) (150 nM) (C, F, I, L, O) were added to virion/cell mixtures for additional 3 h. 24 h later, micrographs were taken at fluorescence microscope; in a replicate sample, cells were then trypsinized, and the number of fluorescent cells was determined by flow cytometry (figures at the right-hand corner in each panel). (P) The number of infected cells determined by FACS in panels A-O is shown as a histogram.

In a simplistic view, a receptor can mediate virus entry by promoting juxtaposition of the cell and virion membranes. Alternatively, the receptor induces conformational changes and promotes activation of the cognate glycoprotein. Experimentally, the two mechanisms can be differentiated since, in the latter, but not in the former case, a soluble form of the receptor (or of the glycoprotein), can substitute for the membrane-form of the receptor (or of the glycoprotein) [63,64]. The results described in the preceding paragraph hint that HER2 is capable to activate the scFv-gB and scFv-gH chimeras of R-909, and R-809. To verify this further, we asked whether entry of R-909, and R-809, can be mediated by a soluble form of HER2. The receptor-negative J cells were exposed to R-909, R-809, R-LM113 R-LM249 and R-LM5 recombinants in the presence of a soluble form of HER2, or of BSA or soluble nectin1, as controls. Twenty-four h later, cultures were scored by fluorescence microscopy, and the number of fluorescent cells was quantified by FACS. The results of a typical experiment are shown in Fig 9A–9O; the quantification is reported as histogram in Fig 9P, in which the lanes are named with the same letters as the corresponding A-O panels. Soluble HER2 promoted entry of all three sets of recombinants, R-909 (panel N), R-809 (panel K), and R-LM113 and R-LM249 (panels E, H). Soluble nectin1 mediated entry of R-LM5 (panel A), as expected. Infection was negligible for all retargeted viruses in the absence of soluble HER2 (panels D, G, J, M), or in the presence of BSA (panels F, I, L, O). The results clearly indicate that the soluble HER2 was able to promote infection of all HER2-retargeted recombinants, and favour the view that HER2 acts by promoting conformational modifications to the respective HER2-retargeted glycoproteins.

## Discussion

In this study we addressed the question whether gB is a suitable glycoprotein for HSV retargeting. The aim was twofold, i.e., to better understand the biology of HSV entry through the lesson of retargeting studies, and to expand and improve the toolkit for the design of retargeted oncolytic HSVs. We report on three findings. First, the insertion of a scFv to HER2 in gB re-addresses HSV tropism to the HER2 receptor. In essence, when gB carries a ligand, the ligand receptor contributes to define the host range of the virus. This was an unexpected result since gB is responsible to execute the virion-cell fusion in HSV and across the *Herpesviridae* family. Second, we asked how does the novel HER2 receptor enable entry of the recombinant carrying the HER2-retargeted gB. We deleted a portion of gD, so that the virus lost the ability to use and be activated by the natural gD receptors; we further depleted cells of β6 and β8 integrins, which activate gH. We found that the entry of the gB-retargeted recombinant occurred in the absence of the cascade of gD and gH activation. Third, retargeting through the chimeric gB_HER2_ was intriguing since, the same scFv ligand to HER2 mediates entry when engineered in gD [45,46], gH [48], or gB (this work). This raised the question of how can the same receptor—HER2—enable virus entry by targeting alternatively gD, gH or gB, despite the fact that these glycoproteins carry out specific, non-interchangeable functions in wt-virus. We discuss these issues separately.

### gB is an effective tool for HSV tropism retargeting

R-909 is highly specific for HER2-positive cells. It replicated to similar yields as the gD-retargeted R-LM113 and the gH-retargeted R-809. It effectively killed HER2-positive cancer cells. gB expands and improves the toolkit for the design of retargeted oncolytic HSVs. It can be envisioned that retargeting *via* gB could be combined with retargeting *via* gD, or *via* gH, so as to generate oncolytic HSVs capable to target cancer cells heterogeneous in receptor display.

### Why retargeting *via* gB was unexpected

gB is the fusogenic glycoprotein in HSV and across the *Herpesviridae* family. The crystal structure of the post-fusion conformation showed features typical of fusion glycoproteins. gB is a trimer, with a central coiled coil, and a crown that carries binding sites for major neutralizing antibodies [11]. Each monomer carries a bipartite fusion loop. A closely similar structure is exhibited by gB from Epstein Barr virus and human cytomegalovirus, hence the structure is conserved across the *Herpesviridae* family [12,65]. The crystals were obtained for gB alone; no co-crystal of gB with cellular proteins was reported. The structure of the prefusion conformation was inferred recently by electron cryotomography [66].

So far, gB was not recognized to be a determinant of HSV tropism. HSV gB interacts with three receptors, PILRα, myelin associated glycoprotein, and non-muscle myosin heavy chain IIA and IIB [27–30]. However, the role played by these receptors in HSV entry, including their contribution to the host range of the virus, is poorly understood. Whether the receptors induce conformational changes to gB, and contribute to gB activation is also unknown. It was noted that the PILRα-mediated entry necessitates of gD [27].

### R-909 entry occurs in the absence of receptor-mediated activation of gD and gH

Two series of experiments support this contention. First, R-909 carries the deletion of AA 6–38 in gD, which ablates its ability to interact with, and be activated by nectin1/HVEM. Hence, R-909 infection occurs in the absence of a nectin1/HVEM-mediated gD activation. Second, infection of R-909, but not of wt-virus, occurred in cells depleted of β6 and β8-integrins [26]. At attachment/entry of R-909, the displacement of gL from gH/gL did not take place. For wt-HSV, gL displacement appears to be part of the process of gH activation [32]. We conclude that R-909 infection occurs in the absence of integrins-mediated gH activation. Altogether, whereas entry of the wt-virus requires the activation of gD and of gH by their respective receptors in a cascade fashion, entry of the gB-retargeted virus does not. HER2 directly activates the chimeric gB. We conclude that the chimeric gB carries two functional domains: the scFv that enables gB activation upon binding to HER2, and the fusogenic domain, intrinsic to the glycoprotein.

With respect to gD, earlier and current work hinted that gD may encode activities other than triggering of glycoprotein activation upon receptor binding [42,48,54,55]. We reasoned that, since the entry glycoproteins assemble in complexes [14–17], even in resting virions [17], the complete absence of anyone of the glycoproteins, or their binding to high amounts of antibodies, may compromise the stability/structure/stoichiometry/gymnastics of the complex, independently of their role in the cascade of activation. We refer to this as a “structural” role of the glycoproteins [48], and searched evidence for it in a cell-cell fusion assay. The gB_HER2_, or the gH_HE2_, or their wt counterparts, were transfected in combination with other components of the fusogenic quartet (gD, gH, gL, gB). The transfected cells were allowed to fuse with target cells expressing HER2 or nectin1. In all combinations the fusion by HER2-retargeted gB, HER2-retargeted gH, or their wt counterparts required gD, be it wt or the non-activable gD_Δ6–38_. This provides formal evidence for gD activity other than triggering, most likely for a structural role.

### How can the same receptor—HER2—enable entry by targeting alternatively gD, gH, or gB?

**Fig 10:**
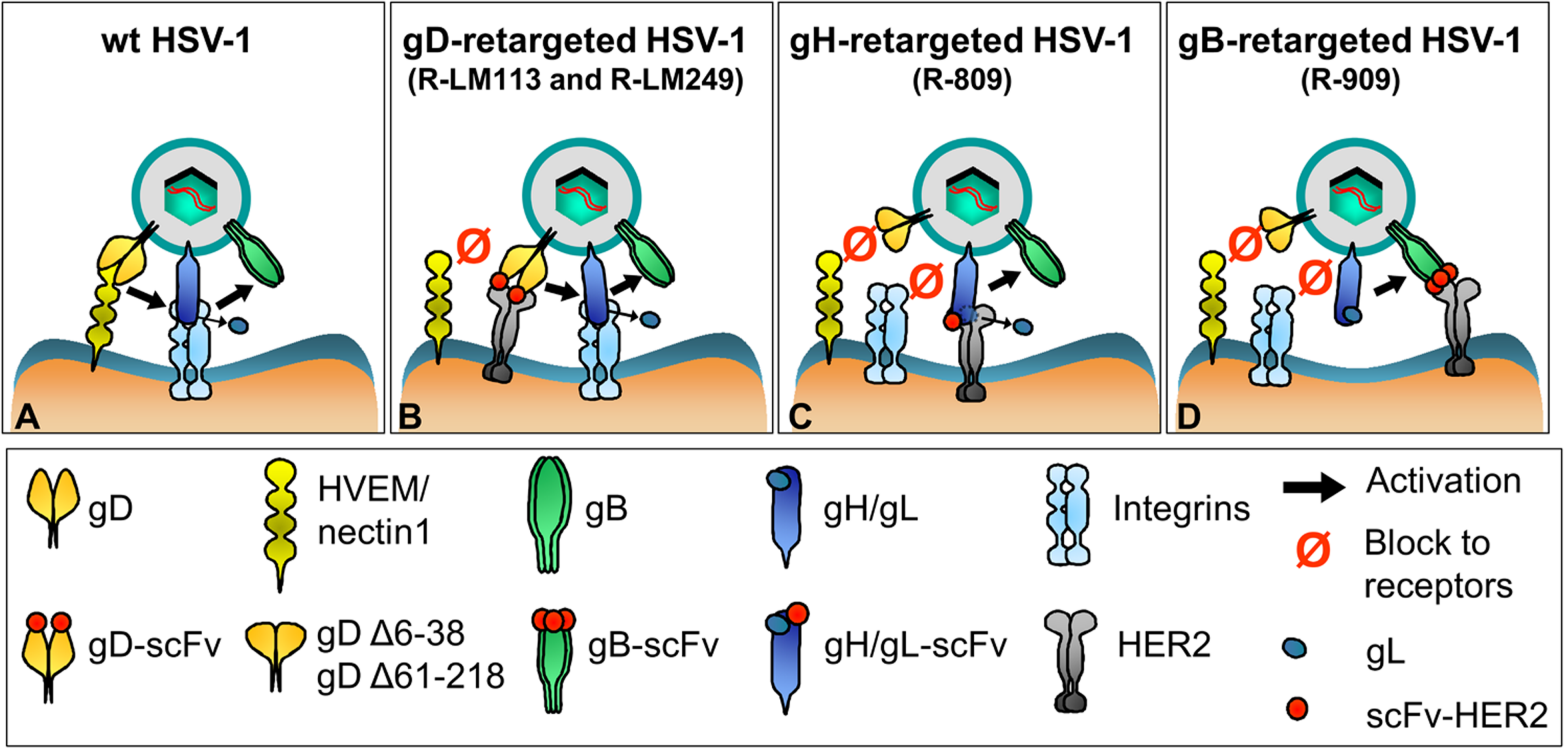
Schematic entry pathways of HER2-retargeted recombinants and of wt-HSV-1. (A) wt-HSV. HVEM, or nectin1-activated gD activates gH/gL, which upon additionally activation by integrins releases gL, and likely transmits signal to gB. (B) gD-retargeted HSVs, R-LM113 and R-LM249. The HER2-retargeted gD is activated by HER2 and not by nectin1/HVEM. gH activation by activated gD, release of gL, and signal transmission to gB is as in wt-virus. (C) gH-retargeted R-809. The chimeric scFv-HER2-gH is activated directly by HER2, and not by gD, nor by integrins. gL is released. HER2-activated gH signals to gB. (D) gB-retargeted R-909. The chimeric scFv-HER2-gB is activated directly by HER2, and not by gD, gH, and integrins. gL is not released.

To shed further light on the mechanism of HER2-mediated entry, we compared three sets of recombinants: the gD-retargeted R-LM113 and R-LM249, the gH-retargeted R-809, and the gB-retargeted R-909. For all three recombinants the soluble HER2 was able to replace the membrane-bound HER2 in mediating entry. Thus, a common feature was that HER2 did not serve the function of anchoring the viruses to the cell; rather it induced conformational modifications to the respective targeted glycoprotein. Interestingly, HER2 exerted differential effects on the entry apparatus of the three sets of recombinants. While the wt R-LM5 required nectin1/HVEM for gD activation and integrin for gL displacement, the gD-retargeted viruses required HER2 for gD activation, and integrins for gH activation and gL displacement (Fig 10). In the case of the gH-retargeted R-809, HER2 brought about both gH activation and gL displacement; gD activation by nectin1/HVEM was not needed. In the case of the gB-retargeted R-909, neither the gD nor gH activation and gL displacement were needed (Fig 10). HER2 directly activated gB: because of this direct activation, there was no need for integrins and for gL release. Thus, the direct activation of the chimeric gB_HER2_ by HER2 bypassed the need for activation by receptor-activated gD and gH.

### Concluding remark

It is possible to engineer a chimeric gB which carries two topologically and functionally distinct domains: the scFv that enables gB activation upon binding to the HER2, and the fusogenic machine, intrinsic to the glycoprotein. The chimeric gB does not need the activation signaling by receptor-bound gD and gH. Functionally, the entry machine of the recombinant R-909 is a monopartite apparatus.

The question arises as to why herpesviruses evolved to have a multipartite entry system. The advantages of a multipartite entry system were in part addressed in a recent review (see, [62]). Briefly, HSV needs specific integrins in order to promote endocytosis, the preferred route of entry for HSV and for other herpesviruses [26,60,67]. For most of the retargeted HSVs, this role appears to be carried out by HER2, which promoted the endocytic pathway of entry. In addition, the multipartite system allows the virus to synchronize endocytosis with the cascade of activation of the glycoproteins, so as to avoid premature activation, and exhaustion of the entry apparatus [32,62]. Integrins activate a signalosome, which can be usurped to the benefit of the virus. Across the family, the multipartite system may ensure a broad and diversified spectrum of receptors [62]. From an evolutionistic point of view, the monopartite system may not ensure the level of sophistication granted to the family by the multipartite system.

## Materials and methods

### Cells and viruses

The J cells **(**negative for HSV receptors) and their derivatives expressing HER2, nectin1 or HVEM were previously described [44,68]. J cells were in turn derived from baby hamster kidney tk- (BHKtk-) cells (line B-1, GM0348A; National Institute of General Medical Sciences Human Genetic Mutant Cell Repository, Bethesda, Md.), described in [69]. The R6 cell line is a derivative of rabbit skin (RS) cells which expresses HSV gD in inducible manner, and can complement HSV mutants in gD [70]. The RS cells were a generous gift from Prof. Bernard Roizman (University of Chicago), who in turn received them from Dr. J. McLaren (University of New Mexico) [71]. The above cells were grown in DMEM (#31600–083, Gibco Laboratories) supplemented with 5% fetal bovine serum (FBS) (#10270–106—E.U.-approved, South America origin, Gibco Laboratories). The CHO, MDA-MB-231, MDA-MB-453, BT-474, SK-OV-3, HaCaT and HeLa cells were purchased from ATCC and cultured as recommended by ATCC. F-12 (Ham) nutrient mixture medium (#BE12615F, Lonza Group Ltd.) (CHO cells), Dulbecco’s modified Eagle medium (#31600–083, Gibco Laboratories), RPMI 1640-Glutamax (#61870010, Gibco Laboratories) and FBS (#10270–106) were supplied by Lonza Group Ltd. or GIBCO Laboratories (Life Technologies, Milano) as specified. The HSV-1(F) received from Dr. B. Roiman was the prototype wt virus [72]. The recombinant viruses R-LM5, R-LM113, R-LM249, R-803 and R-809 were described elsewhere [45,46,48].

### Antibodies and soluble receptors

R8 polyclonal antibody (PAb) to gD and BD80 monoclonal antibody (MAb) to gD were generously provided by Dr. G. H. Cohen and Dr. R. Eisenberg. MAbs HD1 and H126 were a gift from Dr. L. Pereira. MAb H1817 was purchased from Goodwin Institute. MAbs 52S and 53S were described [52]. A PAb to gH/gL was derived to a soluble form of gH truncated at aa 789/gL produced in insect cells [17]. Recombinant human ErbB2/HER2 Fc chimera corresponding to soluble form of HER2 (S-HER2) was purchased from R&D System. Soluble form nectin1 (S-Nectin1) was described [68]. Bovine serum albumin (BSA) was purchased from Sigma-Aldrich.

### Engineering of HSV recombinants expressing genetically modified gBs

First, we engineered R-903 by insertion of the sequence encoding the trastuzumab scFv between AA 43 and 44 of immature gB, corresponding to AA 13 and 14 of mature gB, after cleavage of the signal sequence, which encompasses AA 1–30. The starting genome was the BAC LM5 BG, which carries LOX-P-bracketed pBeloBAC11 and eGFP sequences inserted between UL3 and UL4 of HSV-1 genome [45]. The engineering was performed by galK recombineering. Briefly, the galK cassette with homology arms to gB was amplified by means of primers gB43GalKfor GGTGGCGTCGGCGGCTCCGAGTTCCCCCGGCACGCCTGGGGTCGCGGCCGCGCCTGTTGACAATTAATCATCGGCA and gB43GalKrev GGCCAGGGGCGGGCGGCGCCGGAGTGGCAGGTCCCCCGTTCGCCGCCTGGGTTCAGCACTGTCCTGCTCCTT using pGalK as template. This cassette was electroporated in SW102 bacteria carrying LM55 BG BAC. The recombinant clones carrying the galK cassette were selected on plates containing M63 medium (15 mM (NH_4_)_2_SO_4_, 100 mM KH_2_PO_4_, 1.8 μg FeSO_4_·H_2_O, adjusted to pH7) supplemented with 1 mg/L D-biotin, 0,2% galactose, 45 mg/L L-leucine, 1 mM MgSO_4_·7H_2_O and 12 μg/ml chloramphenicol. In order to exclude galK false positive bacterial colonies, the positive recombinant clones were streaked also on MacConkey agar base plates supplemented with 1% galactose and 12 μg/ml chloramphenicol and checked by colony PCR with primer galK_129_f ACAATCTCTGTTTGCCAACGCATTTGG and galK_417_r CATTGCCGCTGATCACCATGTCCACGC. Next, the trastuzumab scFv cassette with the downstream Ser-Gly linker and bracketed by homology arms to gB was amplified with primers gB43_sc4D5_for GGTGGCGTCGGCGGCTCCGAGTTCCCCCGGCACGCCTGGGGTCGCGGCCGCGTCCGATATCCAGATGACCCAGTCCCCG and gB43_sc4D5_rev GGCCAGGGGCGGGCGGCGCCGGAGTGGCAGGTCCCCCGTTCGCCGCCTGGGTACCGGATCCACCGGAACCAGAGCC using pSG-scFvHER2-SG plasmid as template [48]. The recombinant genome encodes for the chimeric gB, which carries the scFv to HER2 and one downstream Ser-Gly linker, with sequence SSGGGSGSGGSG, and one linker between VL and VH region with the sequence SDMPMADPNRFRGKNLVFHS. The recombinant clones carrying the excision of the galK cassette and the insertion of the sequence of choice, scFv-HER2, were selected on plates containing M63 medium (see above) supplemented with 1 mg/L D-biotin, 0.2% deoxy-2-galactose, 0.2% glycerol, 45 mg/L L-leucine, 1 mM MgSO4·7H_2_O and 12 μg/ml chloramphenicol. Bacterial colonies were also checked for the presence of sequence of choice by means of colony PCR with primers gB_ext_for GAGCGCCCCCGACGGCTGTATCG and gB_431_rev TTGAAGACCACCGCGATGCCCT. The starting material for R-909 was the R-903 BAC genome. To engineer the AA 6–38 deletion in gD, galK cassette flanked by homology arms to gD was amplified with primers gD5_galK_f TTGTCGTCATAGTGGGCCTCCATGGGGTCCGCGGCAAATATGCCTTGGCGCCTGTTGACAATTAATCATCGGCA and gD39_galK_r ATCGGGAGGCTGGGGGGCTGGAACGGGTCCGGTAGGCCCGCCTGGATGTGTCAGCACTGTCCTGCTCCTT. Next, we replaced galK sequence with a synthetic double-stranded oligonucleotide gD_aa5_39f TTGTCGTCATAGTGGGCCTCCATGGGGTCCGCGGCAAATATGCCTTGGCGCACATCCAGGCGGGCCTACCGGACCCGTTCCAGCCCCCCAGCCTCCCGAT. To reconstitute the recombinant virus R-903, 500 ng of BAC DNA was transfected into SK-OV-3 cells by means of Lipofectamine 2000 (Life Technologies). Alternatively, for R-909, the corresponding BAC was first transfected in gD-complementing cell line R6 (a rabbit skin cell derivative expressing glycoprotein D under the control of HSV late promoter UL26.5) [70]. After one passage, the infected cells were frozen. R-909 was subsequently grown and titrated in SK-OV-3 cells. Virus growth was monitored by green fluorescence. The recombinants were genotyped by sequencing the gB, gD and gH ORFs. To detect the expression of gB_HER2_, SK-OV-3 cells were infected at 3 PFU/cell with R-909 and R-LM5, and harvested 72 h after infection. Cell lysates were subjected to SDS-PAGE, transferred to polyvinylidene difluoride membranes and immunoblotted with MAb H1817 to gB.

### Infection of J cells expressing single receptors and of HER2^+^ and HER2^-^ cancer cells

The indicated J cell derivatives were infected with R-909, R-903, R-809 and R-LM5 at an input multiplicity of infection of 3 PFU/cell for 90 min at 37°C and monitored by fluorescence microscopy 24 h post infection. The SK-OV-3, BT-474, MDA-MB-453 HER2-pos cancer cells, and theHER2-neg HeLa and MDA-MB-231 cancer cells, and the HER2-neg non-cancer HaCaT cells were infected at an input multiplicity of infection of 3 PFU/cell (as titrated in SK-OV-3) for 90 min at 37°C with R-909 and R-903. Pictures were taken 24 h after infection by fluorescence microscopy.

### Virus growth and plaque formation

SK-OV-3 cells were infected at 0.1 PFU/cell for 90 min at 37°C. Unabsorbed virus was inactivated by means of an acidic wash (40 mM citric acid, 10 mM KCl, 135 mM NaCl, pH 3). Replicate cultures were frozen at the indicated times (3, 24 and 48 h) after infection and the progeny was titrated in SK-OV-3. For plaque size determinations, the indicated viruses were plated onto SK-OV-3 cells with agar overlay. Pictures of 10 plaques were taken for each virus. Plaque areas (pxE2) were measured with Nis Elements-Imaging Software (Nikon). Each result represents average areas ± SD.

### Cell viability assay

SK-OV-3, MDA-MB-453 and MDA-MB-231 cells were seeded in 96 well plates 8 x 10^3^ cells/well, and infected with the indicated viruses or mock-infected for 90 min at 37°C. The input multiplicity of infection (as titrated in the correspondent cell line) was 2 PFU/cell for the SK-OV-3 and MDA-MB-453. MDA-MB-231 cells were infected with the recombinant viruses R-909 and R-809 at approximately 0.1 PFU/cell (in these cells the viruses do not form plaques, but only singly infected cells). MDA-MB-231 cells were infected with the wt HSV-1(F) at 0.05 PFU/cell. AlamarBlue (Life Technologies) was added to the culture media (10 μl/well) at the indicated times after virus exposure and incubated for 4 h at 37°C. Plates were read at 560 and 600 nm with GloMax Discover System (Promega). For each time point, cell viability was expressed as the percentage of AlamarBlue reduction in infected *versus* uninfected cells, after subtraction of the background value (medium alone). Each point represents the average of at least three triplicate samples ± SD.

### Binding of chimeric glycoproteins to soluble form of HER2

100 ng of DNA encoding wt gH/gL, gH_HER2_/gL, wt gB or gB_HER2_ was transfected into 293T cells by means of Lipofectamine 2000 (Life Technologies). 24 h later cells were fixed with paraformaldehyde, incubated with a soluble truncated form of HER2 tagged with 6x His tag (10 μg/ml, recombinant Human ErbB2/Her2 Fc chimera, R&D SYSTEM), subsequently incubated with mouse anti-His antibody (1 μg/ml, Sigma-Aldrich), and with Anti-Mouse IgG (Fc specific)-Alkaline Phosphatase (1:3000, Sigma-Aldrich) in presence of BCIP (5-bromo-4-chloro-3-indolyl-phosphate, 166 μg/ml, Sigma-Aldrich) and NBT (nitro blue tetrazolium, 333 μg/ml, Sigma-Aldrich) substrate.

### Block of infection with MAbs to HER2, gB, gH or gD

Replicate monolayers of J-HER2 cells, or SK-OV-3 cells in 12-well plates were preincubated with trastuzumab, the MAb to HER2 from which the scFv-HER2 was derived or with non–immune mouse IgG (28 μg/ml final concentration). After 1 h at 37°C, the cells were infected at an input multiplicity of infection of 5 PFU/cell (as titrated in SK-OV-3) with R-909 and R-LM113 or R-LM5. Alternatively, where indicated, virions were pre-incubated with MAb HD1 to gD (1.5 μg/ml, or 30 μg/ml), MAb H126 to gB (ascites fluid, 1:2000), MAb 52S to gH (ascites fluid 1:25) for 1 h at 37°C, and then allowed to adsorb to cells for 90 min. In the case of MAb HD1, the combination of HD1 plus trastuzumab was also tested. Viral inocula were then removed, and cells were overlaid with medium containing the indicated antibodies. Infection was quantified by fluorescent activated cell sorter (FACS).

### Plasmids

Expression plasmids for wt-gD, gB, gH, and gL were described [73]. EGFR2Δ (named erb-2) carries the extracellular domain and transmembrane (TM) sequences of rat HER-2/neu (nucleotides 25 to 2096) (GenBank accession number NM_017003) and is deleted of the tyrosine kinase domain [74]. Plasmid pcagt7 contains the T7 RNA polymerase gene under control of the CAG promoter, and the pt7emcluc plasmid expresses the firefly luciferase under the T7 promoter [75]. Plasmids encoding nectin1 and HER2 were described [21,76].

gB_HER2_, gH_HER2_, and gD_Δ6–38_ were PCR amplified from R-909 or R-803, and cloned into pcDNA3.1(-) (Thermo Fisher Scientific), as follows. gB_HER2_ was amplified with primers gB5_XbaIf CCCCGTAGTTCTAGAGGCACGACGGGCCCCCGTAGTCCCGCCATGC and gBB_EcoRI_r ACAACAAACGAATTCGATGCACATGCGGTTTAACACCCGTGG, then digested with XbaI and EcoRI (New England Laboratories). The 3498 bp XbaI/EcoRI fragment was ligated into the XbaI/EcoRI MCS of pcDNA3.1(-). The gH_HER2_ was amplified with primers gH803_XbaI_f GGGACGGGGTCTAGAATGGGGAATGGTTTATGGTTCG and gH803_NotI_r CCGAAGCCAGCGGCCGCTTATTCGCGTCTCCAAAAAAACGGG, then digested with XbaI and NotI (New England Laboratories). The 3325 bp XbaI/NotI fragment was ligated into XbaI/NotI MCS of pcDNA3.1(-). gD_Δ6–38_ was amplified with primers gD_XbaI_f GTGGTGCGTTCTAGAATGGGGGGGGCTGCCGCCAGG and gD_NotI_r CCATTAAGGGCGGCCGCCTAGTAAAACAAGGGCTGGTGCG, then digested with XbaI and NotI (New England Laboratories). The 1093 bp XbaI/NotI fragment was ligated into XbaI/NotI MCS of pcDNA3.1(-). Inserts were verified by sequencing and by immunofluorescence of the encoded glycoproteins in transfected cells.

### Cell-cell fusion assay

The luciferase-based cell-cell fusion assay was performed as described [77,78]. Donor CHO cells were transfected with the indicated glycoprotein mixture, and the targeted CHO cells with the indicated receptor, or no receptor. Detection was done by means of a luciferase assay system (Promega). The total amount of transfected plasmid DNA was made equal by the addition of EGFR2Δ 2 plasmid. Each value represents the average of triplicate samples.

### Inhibition of infection by Bafilomycin A

A 160 mM stock solution of Bafilomycin A (BFLA) (Sigma Aldrich) was prepared in dimethyl sulfoxide, and further diluted in medium. Cells were exposed to BFLA for 1 h at 37°C and then infected with R-LM5, R-LM113, R-LM249, R-809 and R-909 (5 PFU/cell) for 90 min in the presence of BFLA. The viral inoculum was removed and the cells were overlaid with medium containing BFLA for 18 h. The extent of infection of R-809 and of the GFP-expressing viruses was determined by FACS, or by GloMax Discover System (Promega), respectively.

### Integrin silencing and RT-PCR

Integrins were silenced in SK-OV-3 cells by means of ON-TARGET plus, smart polls (Dharmacon), as previously described [26]. Control cells were transfected with siRNA to E.coli-poliA_0054 [26]. Extent of silencing was determined by RT-PCR using TaqMan gene expression assay (Applied Biosystems).

### Flow cytometry and analysis of media

J cells transfected with indicated receptors were exposed for 30 min at 37°C to R-LM5, R-LM113, R-LM249, R-809, and R-909 at 10 PFU/cell, fixed with 4% (wt/vol) paraformaldehyde and incubated with 53S or 52S MAbs and secondary antibody. At the end of virus absorption, the culture medium was concentrated; devoid of serum IgGs by Protein A-Sepharose and subjected to SDS/PAGE. Immunoblot was performed by means of PAb to gH/gL, MAb H1817 to gB and MAb BD80 to gD.

### Entry mediated by soluble form of HER2

The receptor-negative J cells were exposed to R-LM5, R-LM113, R-LM249, R-809 and R-909 for 3 h at 37°C. Soluble form of HER2 (150 nM) and bovine serum albumin (150 nM), used as negative control, were added to cell-virions mixture for additional 3 h. As positive control, soluble nectin1 (150 nM) was added to J cells exposed to R-LM5. 24 h later pictures were taken by fluorescence microscopy and the number of fluorescent cells was determined by flow cytometry.
